# Transcriptomic Signatures in Sepsis and a Differential Response to Steroids. From the VANISH Randomized Trial

**DOI:** 10.1164/rccm.201807-1419OC

**Published:** 2019-04-15

**Authors:** David B. Antcliffe, Katie L. Burnham, Farah Al-Beidh, Shalini Santhakumaran, Stephen J. Brett, Charles J. Hinds, Deborah Ashby, Julian C. Knight, Anthony C. Gordon

**Affiliations:** ^1^ Section of Anaesthetics, Pain Medicine and Intensive Care, Department of Surgery and Cancer, Faculty of Medicine, Imperial College London, London, United Kingdom; ^2^ Centre for Perioperative and Critical Care Research, Imperial College Healthcare National Health Service Trust, London, United Kingdom; ^3^ Wellcome Centre for Human Genetics, Nuffield Department of Medicine, University of Oxford, Oxford, United Kingdom; ^4^ Imperial Clinical Trials Unit, Faculty of Medicine, Imperial College London, London, United Kingdom; and; ^5^ Intensive Care Unit, Barts and the London, Queen Mary School of Medicine, London, United Kingdom

**Keywords:** sepsis, norepinephrine, vasopressin, corticosteroids, transcriptomics

## Abstract

**Rationale:** There remains uncertainty about the role of corticosteroids in sepsis with clear beneficial effects on shock duration, but conflicting survival effects. Two transcriptomic sepsis response signatures (SRSs) have been identified. SRS1 is relatively immunosuppressed, whereas SRS2 is relatively immunocompetent.

**Objectives:** We aimed to categorize patients based on SRS endotypes to determine if these profiles influenced response to either norepinephrine or vasopressin, or to corticosteroids in septic shock.

**Methods:** A *post hoc* analysis was performed of a double-blind, randomized clinical trial in septic shock (VANISH [Vasopressin vs. Norepinephrine as Initial Therapy in Septic Shock]). Patients were included within 6 hours of onset of shock and were randomized to receive norepinephrine or vasopressin followed by hydrocortisone or placebo. Genome-wide gene expression profiling was performed and SRS endotype was determined by a previously established model using seven discriminant genes.

**Measurements and Main Results:** Samples were available from 176 patients: 83 SRS1 and 93 SRS2. There was no significant interaction between SRS group and vasopressor assignment (*P* = 0.50). However, there was an interaction between assignment to hydrocortisone or placebo, and SRS endotype (*P* = 0.02). Hydrocortisone use was associated with increased mortality in those with an SRS2 phenotype (odds ratio = 7.9; 95% confidence interval = 1.6–39.9).

**Conclusions:** Transcriptomic profile at onset of septic shock was associated with response to corticosteroids. Those with the immunocompetent SRS2 endotype had significantly higher mortality when given corticosteroids compared with placebo.

Clinical trial registered with www.clinicaltrials.gov (ISRCTN 20769191).

**At a Glance Commentary**

**Scientific Knowledge on the Subject**

Several studies investigating corticosteroids in septic shock have found beneficial effects on shock duration. However, effects on survival are varied, with some studies reporting a survival advantage but others finding no benefit. The role of corticosteroids in septic shock remains uncertain, and the reasons for the variation in study outcomes remain unclear. Recently, two transcriptomic sepsis response signatures (SRSs) have been associated with immune function and outcome in sepsis.

**What This Study Adds to the Field**

This is the first study to examine, in septic shock, the interaction between SRS endotypes, and the response to norepinephrine or vasopressin, or to corticosteroids, in the context of a randomized trial. Although SRS endotype had no influence on mortality from sepsis based on vasopressor choice, there was a significant interaction between SRS endotype and treatment with hydrocortisone with regard to mortality. Patients with the immunocompetent SRS2 endotype who were treated with corticosteroids had poorer survival than those given placebo. SRS endotype at the onset of septic shock appears to influence response to corticosteroids. This finding may account for the variation in survival benefit attributed to corticosteroids if varying proportions of the SRS endoypes were recruited into previous trials.

Sepsis is defined as life-threatening organ dysfunction due to a dysregulated host response to infection (1), and is a major global health problem. Current treatment of septic shock relies on antibiotics, fluids, and vasopressors. No new or specific treatments for sepsis are in routine clinical practice. Corticosteroids have been proposed as an adjunctive treatment for septic shock. However, results of clinical trials have been contradictory regarding their impact on outcomes. Recently, two large clinical trials have been published examining the effect of corticosteroids on mortality in septic shock. The ADRENAL (Adjunctive Glucocorticoid Therapy in Patients with Septic Shock) study (2) compared a hydrocortisone infusion to placebo, whereas APROCCHSS (Hydrocortisone Plus Fludrocortisone for Adults with Septic Shock) (3) used a combination of hydrocortisone and fludrocortisone. Both showed clear benefits of corticosteroids on cardiovascular outcomes, demonstrated by shorter durations of shock; however, the effects on survival were inconsistent, with APROCCHSS reporting improved survival with corticosteroid treatment and ADRENAL reporting no difference. We hypothesize that variation in underlying patient phenotypes may account for these differences in outcome.

We have previously identified two transcriptomic sepsis response signatures (SRSs) based on genome-wide expression profiling in patients with sepsis with community acquired pneumonia (4) and fecal peritonitis (5). SRS1 is a relatively immunosuppressed phenotype that is associated with increased mortality, whereas SRS2 is relatively immunocompetent.

In this report, we describe the stratification of patients enrolled into the VANISH (Vasopressin vs. Norepinephrine as Initial Therapy in Septic Shock) clinical trial (6), comparing norepinephrine to vasopressin with or without hydrocortisone for the treatment of septic shock, based on their SRS endotypes. We aimed to determine if transcriptomic phenotype was associated with response to either norepinephrine or vasopressin, or to corticosteroids.

## Methods

### Study Design and Sample Collection

Full details of patient selection and treatment allocation can be found in the online supplement. Patients were recruited into the VANISH trial as previously described (6, 7). The VANISH trial was a factorial (2 × 2), multicenter, double-blind, randomized clinical trial conducted in 18 intensive care units in the United Kingdom between February 2013 and May 2015, with a primary outcome of kidney failure–free days up to Day 28. The trial was approved by the Oxford A research ethics committee, and written consent was obtained from patients or their legal representatives. Adults with septic shock and who required vasopressors were eligible for the trial and were recruited within 6 hours of the onset of septic shock. Patients were randomized to receive either a blinded infusion of vasopressin or norepinephrine (study drug 1), this was titrated to maintain the target mean arterial pressure. Only if the maximum infusion rate of study drug 1 was reached did patients receive the blinded study drug 2, either hydrocortisone or placebo, as previously reported (8). Blood samples for RNA analysis were collected on the day of enrolment into the trial in 10 centers when research staff members were available, and RNA was extracted as described in the online supplement.

### Outcomes

The primary outcome for this analysis was survival at 28 days. Secondary outcomes were kidney failure–free days up to Day 28, intensive care unit and hospital mortality, rates of kidney failure, weaning from vasopressors for greater than 24 hours, time to shock reversal, duration of mechanical ventilation, and mean total Sequential Organ Failure Assessment score (SOFA).

### Statistical Analysis

Patients were allocated to either SRS1 or SRS2 using a generalized linear model based on the set of seven genes (*DYRK2*, *CCNB1IP1*, *TDRD9*, *ZAP70*, *ARL14EP*, *MDC1*, and *ADGRE3*) derived from the previous study of patients with sepsis due to community acquired pneumonia (4, 5). Differential expression analysis was performed using the limma R package (9). For the primary outcome, SRS endotype and drug interaction was explored using binary logistic regression with an interaction term, and differences in survival were displayed using Kaplan-Meier curves using log-rank tests for significance, and Renyi tests when survival curves crossed. As numbers were low in some treatment subgroups, the analysis was repeated using exact logistic regression analysis as a sensitivity analysis (10). As randomization was not stratified by SRS endotype and RNA was only analyzed in a sample of patients, there may be imbalances of potential confounders (age, sex, acute illness score [APACHE (Acute Physiology and Chronic Health Evaluation) II score]), and comorbidities (ischemic heart disease, severe chronic obstructive pulmonary disease, chronic renal failure, cirrhosis, cancer, immunosuppression, and diabetes), so multivariable logistic regression was performed as a sensitivity analysis (5). For both comparisons, vasopressin versus norepinephrine and hydrocortisone versus placebo, only patients who received the study drug as allocated were included, as described in the per-protocol analysis in the primary analysis (6). Further details of the statistical analysis can be found in the online supplement.

## Results

**Figure 1.**
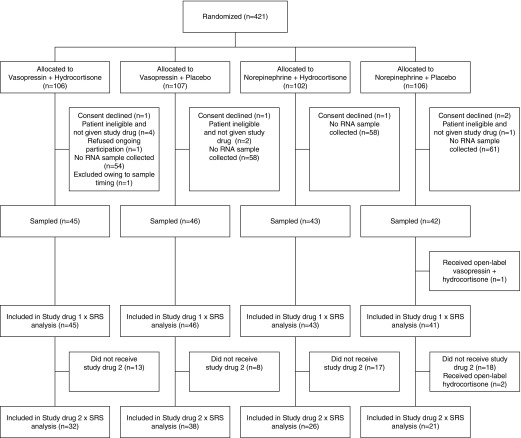
Recruitment, randomization, treatment allocation, and RNA sampling in the VANISH (Vasopressin vs. Norepinephrine as Initial Therapy in Septic Shock) clinical trial. SRS = sepsis response signature.

**Figure 2.**
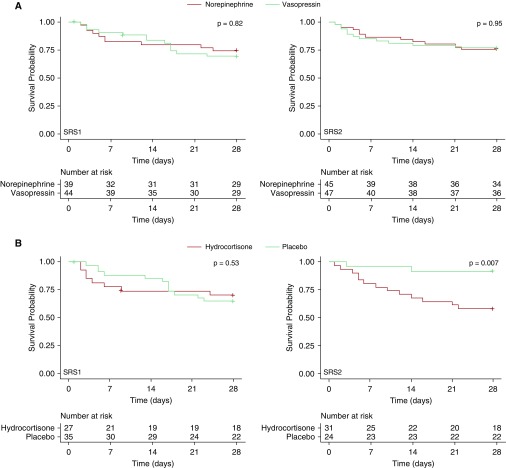
Kaplan-Meier survival curves comparing survival with (*A*) norepinephrine (red line) and vasopressin (green line) and (*B*) hydrocortisone (red line) and placebo (green line) in sepsis response signature (SRS) 1 and SRS2. Crosses represent censored patients (*n* = 2 for SRS1 vasopressin, *n* = 1 for SRS1 placebo, and *n* = 1 for SRS1 hydrocortisone; all other patients were censored at death or Day 29).

Samples were available from 177 patients, Figure 1, but 1 patient was excluded, as the timing of the blood sample was recorded as 9 days after inclusion. The baseline characteristics of these patients were similar to the total trial population, and to those who did not have RNA sampling (*see* Table E1 in the online supplement). The 28-day mortality was also similar in those who did (27%) and did not (31%) have RNA samples taken (*P* = 0.43). Among these 176 patients, 83 (47%) were classified as SRS1 and 93 (53%) as SRS2. We compared global gene expression differences between the SRS endotypes in the VANISH patients to those observed in the derivation study of sepsis due to community-acquired pneumonia (4). We found that SRS, rather than study cohort, was the major driver of the observed variation in gene expression (Figure E1A), and that the differential gene expression results were strongly correlated (Pearson’s *r* = 0.858, *P* < 2.2 × 10^−16^; Figure E1B). Patients with SRS1 and SRS2 endotypes were similar with regard to demographics and baseline characteristics, with only a small difference in rates of ischemic heart disease (higher in SRS2) and serum lactate (higher in SRS1) (Table 1). Baseline characteristics were also similar when patients were stratified by SRS and treatment allocation (Table E2). The effect of vasopressor treatment on mortality at 28 days did not differ statistically between SRS groups (vasopressin vs. norepinephrine in SRS1, odds ratio [OR] = 1.50, 95% confidence interval [CI] = 0.58–3.88; SRS2, OR = 0.94, 95% CI = 0.36–2.46; interaction *P* = 0.50). However, in those patients who received the second study drug, either hydrocortisone or placebo, there was a statistically significant interaction between treatment and SRS endotype (hydrocortisone vs. placebo in SRS1, OR = 0.85, 95% CI = 0.30–2.43; SRS2, OR = 7.9, 95% CI = 1.6–39.9; interaction *P* = 0.02). Kaplan-Meier survival curves are shown in Figure 2. Similar results were obtained using exact logistic regression (hydrocortisone vs. placebo in SRS1, OR = 0.85, 95% CI = 0.26–2.73; SRS2, OR = 7.67, 95% CI = 1.45–78.8; interaction *P* = 0.046). After adjustment for age, sex, disease severity (APACHE II), and comorbidities in multiple logistic regression, hydrocortisone use continued to be associated with increased mortality in those with an SRS2 phenotype (adjusted OR = 8.3, 95% CI = 1.4–47.8), and the treatment by SRS endotype interaction remained significant (interaction *P* = 0.03). In patients who received placebo, mortality was lower in those with the SRS2 compared with SRS1 endotype (unadjusted OR = 0.15, 95% CI = 0.03–0.76, *P* = 0.02; adjusted OR = 0.13, 95% CI = 0.02–0.74, *P* = 0.02; Figure E2), consistent with mortality differences associated with SRS endotypes reported previously (4, 5). Rates and duration of renal failure, and proportions of patients successfully weaned off vasopressors were similar based on SRS and study drug 2 combination (Table 2). Within both SRS1 and SRS2, those patients given hydrocortisone tended to be weaned more quickly from vasopressors (SRS1, HR = 1.3, 95% CI = 0.8–2.3; SRS2, HR = 1.1, 95% CI = 0.6–1.9), although the CIs clearly include 1. Rates of all serious adverse events in the study were the same between SRS endotypes (6 [7%] SRS1 vs. 6 [6%] SRS2, *P* = 0.84).

**Table 1.** Comparison of Baseline Characteristics of Patients with Sepsis Response Signature 1 and 2 Phenotypes

Characteristics	SRS1	SRS2	*P* Value
*n*	83	93	—
Age, median (IQR), yr	66 (53–78)	63 (53–75)	0.40
Men, *n*/total (%)	55/83 (66)	54/93 (58)	0.26
Weight, median (IQR), kg	75 (65–88)	74 (61–92)	0.72
BMI, median (IQR)	26 (23–31)	27 (22–32)	0.68
White race, *n*/total (%)	70/83 (84)	74/93 (80)	0.41
Recent surgical history, *n*/total (%)	15/83 (18)	12/93 (13)	0.34
APACHE II score, median (IQR)	23 (20–30)	24 (19–31)	0.70
Preexisting conditions, *n*/total (%)			
Ischemic heart disease	8/83 (10)	21/93 (23)	**0.02**
Severe COPD	5/83 (6)	5/93 (5)	0.85
Chronic kidney failure	4/83 (5)	4/93 (4)	0.87
Cirrhosis	3/83 (4)	8/93 (9)	0.17
Cancer	12/83 (14)	10/93 (11)	0.46
Immunocompromised	7/83 (8)	3/93 (3)	0.14
Diabetes	16/83 (19)	24/93 (26)	0.30
Organ failure, *n*/total (%)			
Respiratory	33/83 (40)	31/91 (34)	0.44
Kidney	18/83 (22)	22/93 (24)	0.76
Liver	4/73 (5)	8/82 (10)	0.32
Hematological	4/79 (5)	5/92 (5)	0.91
Neurological	27/79 (34)	29/90 (32)	0.79
Physiological variables, median (IQR)			
Mean arterial pressure, mm Hg	70.0 (64.0–76.0)	67.0 (60.5–75.0)	0.16
Heart rate, beats/min	96.0 (85.0–112.0)	92.0 (80.5–104.0)	0.10
Central venous pressure, mm Hg	14 (10–19)	13 (9–18)	0.09
Lactate, mmol/L	2.8 (1.8–4.9)	1.9 (1.3–3.3)	**0.001**
Pa_O_2__/FI_O_2__, mm Hg	197 (122–322)	195 (137–299)	0.96
Creatinine, mg/dl	1.3 (1.0–2.1)	1.4 (0.8–2.3)	0.64
Bilirubin, mg/dl	1.0 (0.5–2.1)	0.7 (0.5–1.3)	0.10
Platelets, ×10^3^/μl	192 (121–267)	187 (120–291)	0.98
GCS	14.0 (6.0–15.0)	13.5 (3.0–15.0)	0.70
Mechanical ventilation, *n*/total (%)	42/83 (51)	54/93 (58)	0.32
Renal replacement therapy, *n*/total (%)	3/83 (4)	2/93 (2)	0.56
Volume of i.v. fluid in previous 4 h, median (IQR), ml	1,255 (547–2,054)	1,003 (557–1,665)	0.09
Patients receiving open-label vasopressor at randomization, *n*/total (%)	72/83 (87)	81/93 (87)	0.95
Time from onset of shock to receiving first study drug, median (IQR), h	4.0 (1.8–5.5)	3.4 (2.0–4.9)	0.44
Norepinephrine dose at randomization, median (IQR), μg/kg/min	0.16 (0.10–0.28)	0.14 (0.08–0.25)	0.25
Source of infection, *n*/total (%)			
Lung	32/82 (39)	45/91 (49)	0.17
Abdomen	21/82 (26)	15/91 (16)	0.14
Soft tissue or line	1/82 (1)	4/91 (4)	0.21
Other	28/82 (34)	27/91 (30)	0.53

*Definition of abbreviations*: APACHE = Acute Physiology and Chronic Health Evaluation; BMI = body mass index; COPD = chronic obstructive pulmonary disease; GCS = Glasgow Coma Score; IQR = interquartile range; i.v. = intravenous; SRS = sepsis response signature.

*P* values are from Mann-Whitney *U* tests for continuous variables and Pearson’s χ^2^ tests for binary variables. Missing data are shown in Table E3 in the online supplement. For the APACHE score, range 0–72, a higher score corresponds to more severe illness and a higher risk of death; for GCS, range 3–15, a lower score corresponds to a greater depression of consciousness; BMI was calculated as weight in kilograms divided by height in meters squared. Bold type indicates *P* < 0.05.

**Table 2.** Comparison of Outcomes of Patients with Sepsis Response Signature 1 and 2 Phenotypes Given Either Hydrocortisone or Placebo as Study Drug 2

	SRS1		SRS2		*P* Value for Interaction
	Hydrocortisone	Placebo	Hydrocortisone	Placebo	
*n*	27	35	31	24	
Kidney failure–free days, median (IQR), d	25 (1–28)	25 (9–28)	25 (4–28)	28 (25–28)	0.43*
28-d mortality, *n*/total (%)	9/27 (33)	13/35 (37)	13/31 (42)	2/24 (8)	**0**.**02**^†^
ICU mortality, *n*/total (%)	7/27 (26)	9/35 (26)	11/31 (35)	2/24 (8)	0.08^†^
Hospital mortality, *n*/total (%)	8/27 (30)	12/35 (34)	13/31 (42)	2/24 (8)	**0**.**02**^†^
Kidney failure, *n*/total (%)	13/27 (48)	19/35 (54)	17/31 (55)	10/24 (42)	0.30^†^
No. weaned from vasopressors for >24 h, *n*/total (%)	25/27 (93)	31/35 (89)	28/31 (90)	23/24 (96)	0.36^†^
Time to shock reversal, median (IQR), h	30.6 (18.1–77.7)	43.8 (21.5–91.5)	58.9 (36.1–82.3)	89.5 (31.5–122.0)	0.60^‡^
Duration of mechanical ventilation, median (IQR), d	3.0 (2.0–12.0)	6.0 (2.0–11.5)	6.0 (2.0–14.5)	9.0 (6.0–20.0)	0.67*
Mean total SOFA score, median (IQR)	5.7 (3.6–9.0)	4.9 (3.6–7.2)	5.6 (3.7–8.3)	4.7 (3.5–6.3)	0.72^§^

*Definition of abbreviations*: IQR = interquartile range; SOFA = Sequential Organ Failure Assessment score; SRS = sepsis response signature.

Bold type indicates *P* < 0.05.

* From the aligned rank transform test.

^†^ From logistic regression.

^‡^ From Cox regression, treating deaths as never having the event of interest. Results were similar treating death as a competing risk.

^§^ From linear regression, applying a square root transform to the outcome.

## Discussion

We were able to identify the two previously identified SRS endotypes within this septic shock population due to diverse etiologies in the VANISH clinical trial. In this study, a higher proportion of patients had the SRS1 endotype (47%) than in either the derivation (41%) or validation (35%) cohorts described in the original study (4). However, the original data were derived from a sepsis population where only a portion had septic shock, with under half requiring vasopressors. Vasopressor use and SOFA score were higher in SRS1 patients in the derivation study, suggesting more severe disease. It is therefore unsurprising that, in a sicker population of patients, all of whom had septic shock, the SRS1 endotype is more commonly represented. Importantly, although the two SRS endotypes have previously been described in both community-acquired pneumonia (4) and fecal peritonitis (5), this is the first time the endotypes have been demonstrated in patients with sepsis due to multiple different sources of infection.

Transcriptomic profile at the onset of septic shock was associated with response to corticosteroids, but not vasopressin or norepinephrine. Those patients with the SRS2 endotype had significantly higher mortality when given corticosteroids compared with placebo. However, this effect on mortality was not seen in those with the SRS1 endotype. Previous work (4, 5) demonstrated that the SRS2 endotype was associated with a significantly lower mortality rate than SRS1. In patients with sepsis with pneumonia, 28-day mortality was 17% in SRS2 compared with 27% in SRS1 (4), and, in fecal peritonitis, 28-day mortality was 7.2% versus 20.8% for SRS2 and SRS1, respectively (5). This pattern was again seen in the current study when only those patients randomized to placebo were considered, where 28-day mortality was lower in SRS2 (8%) compared with SRS1 (37%). As inclusion/exclusion criteria and illness severity vary between different studies, actual mortality rates will inevitably vary.

Steroids have a clear benefit on time-to-shock resolution, reported in multiple clinical trials (2, 3, 8, 11). Despite this improvement in an important physiological measure, the overall effect on patient survival has been inconsistent between trials. Differences in the mortality effects of steroids in the recent ADRENAL (2) and APROCCHSS (3) trials may be explained by the current findings. If these trials recruited different proportions of patients with the two SRS endotypes, a trial with a greater proportion of SRS2 patients may find no survival advantage or may find harm due to steroids in septic shock. In observational studies, SRS1 has been associated with higher mortality than SRS2, and similar effects were seen in placebo patients in this trial. Overall, the mortality in the ADRENAL trial was lower than that seen in the APROCCHSS trial (28% vs. 46%, respectively, at Day 90), perhaps suggesting that a higher proportion of SRS2 patients may have been recruited. If the SRS2 patients are harmed with steroid treatment, it may explain why, overall, no mortality benefit was seen in the ADRENAL trial, despite improvement in shock resolution. Interestingly, the duration of shock tended to be shorter among both SRS1 and SRS2 patients successfully weaned from vasopressors, although the CIs are wide, possibly due to the small numbers in each subgroup.

Because of the many mechanisms of action of corticosteroids, we can only speculate as to why the effect of steroids on mortality should vary between SRS endotypes. The SRS1 endotype has been shown to be a relatively immunosuppressed phenotype with features of endotoxin tolerance, T cell exhaustion, and downregulation of major histocompatibility class (MHC) II antigens, and is associated with higher mortality rates. The SRS2 endotype in contrast is relatively more immunocompetent and associated with lower mortality rates. Of particular interest is the upregulation of MHC II in SRS2 (4). Reduction in HLA-DR expression in sepsis has been associated with higher rates of nosocomial infection and worse survival (12), so it is plausible that improvement in antigen presentation improves immune function and bacterial clearance in the SRS2 group, improving survival compared with SRS1. However, corticosteroids are recognized to downregulate MHC II (12–14), which could provide a mechanism by which this protective advantage is removed. Corticosteroids also have actions affecting NF-κB, T cells, and apoptosis (15, 16), all of which showed evidence of differential expression between the SRS endotypes. Altered modulation of these pathways could account for different degrees of immunosuppression caused by corticosteroids between the SRS endotypes. It is therefore possible that corticosteroids may have beneficial cardiovascular effects in all patients with septic shock, but that the well-known immunosuppressive adverse effects of corticosteroids are only realized in the SRS2 patients. Immune dysfunction is recognized to increase the risk of nosocomial infection and to be associated with higher rates of mortality (17), yet clinical scoring systems, such as SOFA, do not include the immune system. This may account for why no difference in total SOFA score was seen despite mortality differences between treatment groups in our study.

Transcriptomic profiles in sepsis and response to corticosteroid therapy have been studied in children (18). In this previous study, a subgroup of patients was identified using RNA expression that was also associated with worse outcomes when patients were treated with corticosteroids, although this subgroup was the more immunosuppressed phenotype. However, as previously described (5, 19), the gene expression profiles appear to be different in adult and pediatric populations, with those in children being based, in part, on genes linked to glucocorticoid receptors. In the pediatric study corticosteroid treatment was based on physician choice rather than randomized allocation as in this clinical trial.

This study does have limitations. It is a *post hoc* analysis of samples collected as part of a clinical trial. Research blood sampling was not available in all centers, and, due to the emergency nature of the trial and the short recruitment window (maximum 6 h), it was not logistically possible to collect samples from all patients, thus limiting the sample size. However, the subset of patients in this analysis had similar baseline characteristics to the overall trial population, and the result was robust to adjustment for potential confounders and the use of statistical methods to account for small numbers. Although the analysis was *post hoc*, we used predefined endotype definitions based on previously published work and derived and validated in independent cohorts (4, 5). Importantly, treatment allocation was randomized and double blinded. Although the SRS endotypes are described according to their presumed immunological effects, this is based on gene expression data, and the absolute functional implications of the endotypes are still to be established. It is plausible that corticosteroids interact with SRS endotypes in ways that cannot be appreciated from transcriptomic data alone.

Although further work is required to validate these findings and to better understand the utility of endotype assignment based on transcriptomic profiles in sepsis, our findings suggest that SRS endotypes should be used in future biomarker-guided trials of corticosteroids in septic shock.

## Supplementary Material

Supplements

Author disclosures
