# Supplementary material for: Transcriptomic Signatures in Sepsis and a Differential Response to Steroids. From the VANISH Randomized Trial
Source: Am J Respir Crit Care Med. 2019 Apr 15;199(8):980–6. doi: 10.1164/rccm.201807-1419OC (PMC6467319; doi:10.1164/rccm.201807-1419OC)
Supplement: Supplements [file rccm.201807-1419OC.html]

Transcriptomic Signatures in Sepsis and a Differential Response to Steroids. From the VANISH Randomized Trial | American Journal of Respiratory and Critical Care Medicine

- antcliffe\_data\_supplement.pdf (957 KB)
- disclosures.pdf (174 KB)
