# Supplementary material for: Transcriptomic Signatures in Sepsis and a Differential Response to Steroids. From the VANISH Randomized Trial
Source: Am J Respir Crit Care Med. 2019 Apr 15;199(8):980–6. doi: 10.1164/rccm.201807-1419OC (PMC6467319; doi:10.1164/rccm.201807-1419OC)
Supplement: Supplements [file rccm.201807-1419OC_antcliffe_data_supplement.pdf]

Online Data Supplement

Transcriptomic Signatures in Sepsis and a Differential Response to Steroids: From the VANISH Randomized Trial

David B Antcliffe, Katie L Burnham, Farah Al-Beidh, Shalini Santhakumaran, Stephen J Brett, Charles J Hinds, Deborah Ashby, Julian C Knight, Anthony C Gordon

Methods
Table E1: Comparison of baseline characteristics between patients with and without RNA sampling
Table E2: Comparison of baseline characteristics stratified by SRS and treatment allocation
Table E3: Missing data
Figure E1: Comparison of SRS in GAinS and VANISH.
Figure E2: Kaplan-Meier curves comparing survival between SRS1 and SRS2 in those patients randomized to receive placebo and hydrocortisone

Methods

Design

VANISH was a double-blind, factorial (2x2), randomized controlled trial conducted in 18 intensive care units in the United Kingdom.

Treatment Regimen

Patients were randomized to receive blinded vasopressin (0-0·06 U/min) or norepinephrine (0-12 μg/min) as first line vasopressor to maintain the mean arterial blood pressure after adequate fluid resuscitation within the first 6 hours after onset of septic shock. If the maximal dose of the first study drug was reached patients were randomized to receive either hydrocortisone or placebo, the second study drug. If the patient was still hypotensive after the first dose of study drug 2 then open label catecholamine vasopressors could be used. If the patient was still not responding to open label vasopressors then open label IV hydrocortisone could be given as a rescue therapy.

Patients

Inclusion Criteria

Adult patients who required vasopressors for the management of sepsis despite adequate fluid resuscitation.

Patients needed to fulfil the following inclusion criteria:

- Fulfil 2/4 of the systemic inflammatory response syndrome criteria (1) due to known or suspected infection within the previous 24 hours.
- Hypotension despite adequate intravenous fluid resuscitation.

Exclusion Criteria

Patients were excluded if any of the following criteria were met:

- The patient had received a continuous infusion of vasopressors previously during the ICU admission (other than vasopressors used as emergency treatment [for less than six hours] to stabilize the patient during this episode). Vasopressors included norepinephrine, epinephrine, vasopressin, dopamine, metaraminol, phenylephrine, and (intermittent) terlipressin.
- Regular systemic corticosteroid therapy within the previous three months (this did not include inhaled steroid therapy).
- Known adrenal dysfunction / insufficiency.
- End-stage renal failure (i.e. requiring long term dialysis)
- The physician and team were not committed to full active care.
- The patient was known to be pregnant.
- The patient had known acute mesenteric ischemia.
- The patient was known to have Raynaud's phenomenon, systemic sclerosis or other vasospastic diseases.
- The patient had been enrolled in another clinical trial of an investigational medicinal product within 30 days or was enrolled in another interventional study that might interact with the study drugs.
- The patient had a history of anaphylaxis or hypersensitivity to any study drug.

Informed Consent

Due to the emergency nature of the trial, a waiver of initial consent was granted. Patients

could be enrolled without prospective consent and then written consent was obtained from the patient or a personal or professional legal representative as soon as possible. For cases in which a legal representative gave consent, retrospective written consent was sought once the patient regained decision-making capacity.

Randomization

Randomization was carried out by a computer generated random number and was stratified by ICU and occurred on a 1:1:1:1 basis with a variable block size randomization (4 and 8). The allocation sequence was prepared by an independent statistician and concealed from the investigators.

Blood Sampling

Samples were only collected in sites who had the necessary research staff available. 2·5ml of blood was collected in PAXgene tubes (PreAnalytix, Switzerland) for RNA analysis as soon after enrolment into the study as possible. In most cases this was prior to administration of the study drugs but could be up to 12h after drug administration. After blood collection PAXgene tubes were left at room temperature for 45 minutes before being stored at -80ºC.

RNA extraction

RNA was extracted by LGC (Berlin, Germany) using PAXgene RNA kits (Qiagen, Netherlands). RNA was extracted in random batches of 24.

Microarray Analysis

Genome-wide gene expression was quantified using Illumina Human-HT-12 v4 Expression BeadChips (47,323 probes). Raw data were prepared using Illumina’s Genomestudio.

Background signals were subtracted and probes with a detection p-value <0·05 in at least 10% of samples were retained for downstream analysis. The raw data were transformed and normalized using the variance stabilization and normalization method in R (2). Batch differences between chips were corrected using the ComBat package in R (3).

Statistical Analysis

Sepsis Response Signatures (SRS) were previously derived in cohorts of community acquired pneumonia (CAP) (4) and fecal peritonitis (FP) (5) using agglomerative hierarchical clustering on the most variable 10% of probes using Ward's method with Euclidean distance to initiate the appropriate number of groups, followed by *k*-means clustering to consolidate group membership. Plotting the within group sum of squares was used to determine an appropriate number of clusters. Predictors for the SRS groups were selected using GeneRave from differentially expressed genes with moderate to high expression (expression > 6·5) in at least the number of samples in the smaller group. GeneRave uses a sparse regression method to select a minimal number of explanatory variables from a large number of potential predictors.

Patients were allocated to either SRS1 or SRS2 using a generalized linear model based on the set of 7 genes (*DYRK2*, *CCNB1IP1*, *TDRD9*, *ZAP70*, *ARL14EP*, *MDC1,* and *ADGRE3*) derived from the previous study of patients with sepsis due to community acquired pneumonia (4, 5). Baseline characteristics were compared between SRS groups. Categorical variables were compared using Pearson's chi-square test and continuous variables with the Mann-Whitney U test; all were 2-sided tests and a p-value<0·05 was considered significant. Differential expression analysis was carried out using the limma R package (6). For the primary outcome, SRS endotype and drug interaction was explored using binary logistic regression with an interaction term and differences in survival were displayed using Kaplan-

Meier curves using log-rank tests for significance, and Renyi tests when survival curves crossed. As numbers were low in some treatment-subgroups the analysis was repeated using exact logistic regression analysis as a sensitivity analysis (7). As randomization was not stratified by SRS endotype and RNA only analyzed in a sample of patients, there may be imbalances of potential confounders (age, sex, acute illness score [APACHE II score], and comorbidities (ischemic heart disease, severe chronic obstructive pulmonary disease, chronic renal failure, cirrhosis, cancer, immunosuppression, and diabetes) so multivariable logistic regression was carried out as a sensitivity analysis (5). Secondary outcomes were summarized by SRS group and treatment allocation. SRS-treatment interactions were tested using logistic regression for binary outcomes, linear regression for continuous outcomes, and Cox regression for time to event outcomes. Where the assumptions of linear regression were not met, the aligned rank transform test was used (8, 9). Statistical analysis was carried out in R version 3·2 and SPSS version 24 (IBM, USA). In order to assess the effect of SRS endotype on both sets of study drugs, for each comparison, vasopressin vs norepinephrine and hydrocortisone vs placebo, only patients who received the study drug as allocated were included, as described in the per-protocol analysis in the primary analysis (10).

Table E1 Comparison of baseline characteristics between patients with and without RNA sampling

	No RNA Sampling	RNA Sampling	p-value
n	232	177	-
Age median (IQR), years	66 (57-76)	65 (53-77)	0·55
Men, No./total (%)	128/232 (55)	110/177 (62)	0·16
Weight, median (IQR), kg	74·0 (62·5-85·0)	75·0 (62·0-90·0)	0·43
BMI, median (IQR)	25·1 (22·3-29·1)	26·1 (22·6-31·4)	0·17
Caucasian ethnicity, No./total (%)	204/232 (88)	145/177 (82)	0·09
Recent surgical history, No./total (%)	46/232 (20)	27/177 (15)	0·23
APACHE II score, median (IQR)	24·0 (18·5-29·5)	24·0 (19·0-30·0)	0·51
Pre-existing conditions, No./total (%)			
Ischemic heart disease	33/232 (14)	29/177 (16)	0·55
Severe COPD	5/232 (2)	10/177 (6)	0·06
Chronic kidney failure	19/232 (8)	8/177 (5)	0·14
Cirrhosis	4/232 (2)	11/177 (6)	**0·02**
Cancer	25/232 (11)	22/177 (12)	0·60
Immunocompromised	18/232 (8)	10/177 (6)	0·40
Diabetes	50/232 (22)	40/177 (23)	0·80
Organ failure, No./total (%)			
Respiratory	84/227 (37)	65/175 (37)	0·98
Kidney	45/231 (19)	40/177 (23)	0·44
Liver	8/223 (4)	12/156 (8)	0·08
Hematological	13/226 (6)	9/172 (5)	0·82
Neurological	72/215 (33)	56/170 (33)	0·91
Physiological variables, median (IQR)			
Mean arterial pressure, mm Hg	70·0 (62·0-79·0)	68·5 (62·0-75·0)	0·15
Heart rate, beats/min	100 (86-111)	94 (82-109)	**0·02**
Central venous pressure, mm Hg	12 (8-16)	13 (9-19)	0·17
Lactate, mmol/L	2·3 (1·3-4·3)	2.4 (1.5-4.0)	0·46
PaO_2/FiO_2, mm Hg	182 (114-287)	195 (130-309)	0·17
Creatinine, mg/dL	1.4 (0.8-2.5)	1.3 (0.9-2.2)	0·87
Bilirubin, mg/dL	0.9 (0.5-1.5)	0.8 (0.5-1.7)	0·82
Platelets, $\times 10^3/\mu L$	184 (122-290)	192 (121-283)	0·85
GCS	14 (3-15)	14 (5-15)	0·43
Mechanical ventilation, No./total (%)	139/232 (60)	97/177 (55)	0·30
Renal replacement therapy, No./total (%)	6/232 (3)	5/177 (3)	0·88
Volume of IV fluid in previous 4 h, median (IQR), mL	1166 (711-2140)	1067 (549-2000)	0·09
Patients receiving open-label vasopressor at randomization, No./total (%)	194/232 (84)	154/177 (87)	0·34
Time from onset of shock to receiving first study drug, median (IQR), h	3·3 (1·3-5·3)	3·7 (2·0-5·2)	0·11
norepinephrine dose at randomization, median (IQR), mg/kg/min	0·19 (0·12-0·33)	0·15 (0·09-0·27)	**0·02**
Source of infection, No./total (%)			
Lung	87/226 (38)	78/174 (45)	0·20
Abdomen	57/226 (25)	36/174 (21)	0·29
Soft tissue or line	14/226 (6)	5/174 (3)	0·12
Other	68/226 (30)	55/174 (32)	0·74
28 Day Mortality, No./total (%)	71/231 (31)	48/177 (27)	0·43

p-values are from Mann-Whitney U tests for continuous variables and Pearson's χ^2 tests for binary variables

Abbreviations: APACHE, Acute Physiology and Chronic Health Evaluation (range 0-72, a higher score corresponds to more severe illness and a higher risk of death); BMI, body mass index (calculated as weight in kilograms divided by height in meters squared); COPD, chronic obstructive pulmonary disease; GCS, Glasgow Coma Score (range 3-15, a lower score corresponds to a greater depression of consciousness); IQR, interquartile range; IV, intravenous; PaO_2/FIO_2, arterial oxygen partial pressure to fractional inspired oxygen.

Table E2 Comparison of baseline characteristics of patients with SRS1 and SRS2 phenotypes stratified by treatment allocation.

	SRS1		SRS2		SRS1		SRS2	
Drug	Norepinephrine	Vasopressin	Norepinephrine	Vasopressin	Hydrocortisone	Placebo	Hydrocortisone	Placebo
n	39	44	45	47	27	35	31	24
Age median (IQR), y	65 (49-77)	70 (57-79)	63 (55-76)	65 (53-75)	67 (54-79)	70 (52-78)	61 (49-74)	66 (54-75)
Men, No./total (%)	26/39 (67)	29/44 (66)	29/45 (64)	25/47 (53)	19/27 (70)	22/35 (63)	16/31 (52)	14/24 (58)
Weight, median (IQR), kg	75 (68-90)	75 (61-86)	73 (61-94)	75 (62-90)	80 (65-90)	72 (60-85)	75 (62-101)	73 (61-85)
BMI, median (IQR)	26 (23-32)	25 (22-29)	27 (22-31)	27 (23-35)	26 (23-31)	25 (23-29)	28 (23-32)	27 (22-34)
Caucasian ethnicity, No./total (%)	31/39 (79)	39/44 (89)	38/45 (84)	35/47 (74)	22/27 (81)	30/35 (86)	27/31 (87)	16/24 (67)
Recent surgical history, No./total (%)	8/39 (21)	7/44 (16)	5/45 (11)	7/47 (15)	2/27 (7)	10/35 (29)	6/31 (19)	2/24 (8)
APACHE II score, median (IQR)	23 (21-31)	24 (19-30)	26 (19-31)	23 (20-32)	26 (22-35)	25 (21-30)	28 (19-33)	25 (19-32)
Pre-existing conditions, No./total (%)								
Ischemic heart disease	4/39 (10)	4/44 (9)	10/45 (22)	11/47 (23)	4/27 (15)	2/35 (6)	9/31 (29)	3/24 (13)
Severe COPD	2/39 (5)	3/44 (7)	4/45 (9)	1/47 (2)	1/27 (4)	2/35 (6)	1/31 (3)	0/24 (0)
Chronic kidney failure	1/39 (3)	3/44 (7)	1/45 (2)	3/47 (6)	2/27 (7)	2/35 (6)	1/31 (3)	0/24 (0)
Cirrhosis	2/39 (5)	1/44 (2)	2/45 (4)	6/47 (13)	1/27 (4)	2/35 (6)	3/31 (10)	2/24 (8)
Cancer	6/39 (15)	6/44 (14)	5/45 (11)	5/47 (11)	3/27 (11)	6/35 (17)	4/31 (13)	2/24 (8)
Immunocompromised	4/39 (10)	3/44 (7)	0/45 (0)	3/47 (6)	2/27 (7)	4/35 (11)	2/31 (6)	0/24 (0)
Diabetes	10/39 (26)	6/44 (14)	12/45 (27)	12/47 (26)	5/27 (19)	5/35 (14)	8/31 (26)	8/24 (33)
Organ failure, No./total (%)								
Respiratory	18/39 (46)	15/44 (34)	16/45 (36)	14/45 (31)	11/27 (41)	19/35 (54)	14/30 (47)	6/23 (26)
Kidney	8/39 (21)	10/44 (23)	14/45 (31)	7/47 (15)	9/27 (33)	6/35 (17)	8/31 (26)	6/24 (25)
Liver	1/31 (3)	3/42 (7)	5/41 (12)	2/40 (5)	2/26 (8)	1/31 (3)	2/30 (7)	2/19 (11)
Hematological	1/36 (3)	3/43 (7)	2/44 (5)	3/47 (6)	3/27 (11)	1/33 (3)	1/31 (3)	3/23 (13)
Neurological	12/37 (32)	15/42 (36)	14/45 (31)	15/44 (34)	11/27 (41)	13/33 (39)	11/29 (38)	7/23 (30)
Physiological variables, median (IQR)								
Mean arterial pressure, mm Hg	69.5 (64.0-76.5)	70.0 (64.0-75.8)	64.0 (58.5-74.5)	69.0 (62.0-75.0)	69.0 (60.0-78.0)	67.0 (64.0-72.0)	64.0 (59.0-75.0)	65.0 (58.3-71.3)
Heart rate, beats/min	95.0 (84.0-117.0)	97.0 (85.0-110.8)	90.0 (80.0-100.5)	94.0 (81.0-106.0)	92.0 (74.0-110.0)	101.0 (89.0-118.0)	95.0 (84.0-106.0)	84.5 (75.5-102.8)
Central venous pressure, mm Hg	17 (10-20)	13 (10-19)	12 (7-17)	13 (9-19)	16 (10-23)	14 (12-19)	12 (9-18)	16 (11-20)
Lactate, mmol/L	3.1 (1.9-5.9)	2.5 (1.8-4.7)	2.2 (1.4-3.5)	1.8 (1.3-3.2)	3.0 (1.8-5.1)	2.8 (1.7-5.0)	2.4 (1.4-3.4)	2.6 (1.4-3.6)
PaO_2/FiO_2, mm Hg	160 (119-313)	236 (122-338)	199 (150-272)	191 (129-309)	214 (160-313)	153 (98-289)	173 (137-275)	206 (133-269)
Creatinine, mg/dL	1.4 (0.9-2.1)	1.3 (1.0-2.1)	1.5 (0.8-2.7)	1.1 (0.8-2.1)	1.7 (1.4-3.5)	1.2 (0.8-1.5)	1.6 (0.8-3.1)	1.5 (1.0-2.3)
Bilirubin, mg/dL	0.9 (0.4-1.6)	1.0 (0.5-2.7)	0.6 (0.4-1.4)	0.8 (0.5-1.3)	0.8 (0.5-2.1)	0.8 (0.4-1.6)	0.7 (0.4-1.2)	0.8 (0.6-1.7)
Platelets, $\times 10^3/\mu L$	221 (146-324)	160 (108-246)	183 (131-258)	188 (104-312)	208 (118-294)	181 (118-326)	207 (122-308)	207 (104-278)
GCS	14 (3-15)	13 (6-15)	13 (3-15)	14 (3-15)	12.0 (6.0-15.0)	13.0 (3.0-15.0)	13.0 (3.0-15.0)	14.0 (3.0-15.0)
Mechanical ventilation, No./total (%)	22/39 (56)	20/44 (45)	26/45 (58)	27/47 (57)	12/27 (44)	23/35 (66)	21/31 (68)	14/24 (58)
Renal replacement therapy, No./total (%)	1/39 (3)	2/44 (5)	0/45 (0)	1/47 (2)	1/27 (4)	2/35 (6)	1/31 (3)	0/24 (0)
Volume of IV fluid in previous 4 h, median (IQR), mL	1543 (608-2363)	1144 (532-1710)	788 (336-1422)	1168 (812-1760)	1368 (542-2050)	1250 (702-2250)	1200 (731-1750)	1055 (592-1547)
Patients receiving open-label vasopressor at randomization, No./total (%)	33/39 (85)	39/44 (89)	37/45 (82)	43/47 (91)	21/27 (78)	33/35 (94)	28/31 (90)	19/24 (79)
Time from onset of shock to receiving first study drug, median (IQR), h	3.5 (1.8-5.5)	4.1 (2.0-5.5)	3.5 (2.3-4.8)	3.2 (2.0-5.0)	4.3 (1.8-5.5)	3.5 (2.0-5.5)	3.0 (2.0-4.6)	3.2 (2.0-5.3)

Norepinephrine dose at randomization, median (IQR), μg/kg/min	0.16 (0.10-0.25)	0.16 (0.10-0.32)	0.15 (0.08-0.29)	0.14 (0.08-0.22)	0.20 (0.14-0.50)	0.19 (0.11-0.33)	0.16 (0.14-0.38)	0.12 (0.07-0.24)
Source of infection, No./total (%)								
Lung	14/38 (37)	18/44 (41)	21/44 (48)	23/46 (50)	13/26 (50)	14/35 (40)	16/30 (53)	13/24 (54)
Abdomen	14/38 (37)	7/44 (16)	7/44 (16)	8/46 (17)	4/26 (15)	11/35 (31)	6/30 (20)	2/24 (8)
Soft tissue or line	0/38 (0)	1/44 (2)	2/44 (5)	2/46 (4)	1/26 (4)	0/35 (0)	0/30 (0)	3/24 (13)
Other	10/38 (26)	18/44 (41)	14/44 (32)	13/46 (28)	8/26 (31)	10/35 (29)	8/30 (27)	6/24 (25)

Abbreviations: APACHE, Acute Physiology and Chronic Health Evaluation (range 0-72, a higher score corresponds to more severe illness and a higher risk of death); BMI, body mass index (calculated as weight in kilograms divided by height in meters squared); COPD, chronic obstructive pulmonary disease; GCS, Glasgow Coma Score (range 3-15, a lower score corresponds to a greater depression of consciousness); IQR, interquartile range; IV, intravenous; PaO_2/FIO_2, arterial oxygen partial pressure to fractional inspired oxygen.

Table E3 Missing data, given as number of missing values (%)

	Missing Values
n	176
Age	0
Sex	0
Weight	0
BMI	0
Ethnicity	0
Recent surgical history	0
APACHE II score	1 (1)
Pre-existing conditions	
Ischemic heart disease	0
Severe COPD	0
Chronic kidney failure	0
Cirrhosis	0
Cancer	0
Immunocompromised	0
Diabetes	0
Organ failure	
Respiratory	2 (1)
Kidney	0
Liver	21 (12)
Hematological	5 (3)
Neurological	7 (4)
Physiological variables	
Mean arterial pressure	1 (1)
Heart rate	0
Central venous pressure	76 (43)
Lactate	4 (2)
PaO_2/FiO_2	6 (3)
Creatinine	0
Bilirubin	21 (12)
Platelets	5 (3)
GCS	7 (4)
Mechanical ventilation	0
Renal replacement therapy	0
Volume of IV fluid in previous 4 h	2 (1)
Patients receiving open-label vasopressor at randomization	0
Time from onset of shock to receiving first study drug	0
Source of infection	
Lung	3 (2)
Abdomen	3 (2)
Soft tissue or line	3 (2)
Other	3 (2)
28 Day Mortality	0

Abbreviations: APACHE, Acute Physiology and Chronic Health Evaluation; BMI, body mass index; COPD, chronic obstructive pulmonary disease; GCS, Glasgow Coma Score; IQR, interquartile range; IV, intravenous; PaO_2/FIO_2, arterial oxygen partial pressure to fractional inspired oxygen.

Figure E1. Comparison of SRS in GAinS and VANISH. A) Principal component analysis of the top 10% most variable probes for the GAinS patients in which the SRS model was derived (4) and the VANISH clinical trial patients. SRS assignment based on the 7-gene set is indicated by color, and dataset by the point type.
B) Correlation plot showing the log fold change of differential expression of all transcripts between SRS groups in the VANISH cohort compared to the log fold changes for the same genes in the GAinS cohort. Blue coloring indicates significant differential expression in both datasets, green in GAinS only, yellow in VANISH only, and grey indicates the gene is not significantly differentially expressed in either dataset. DE: Differentially Expressed

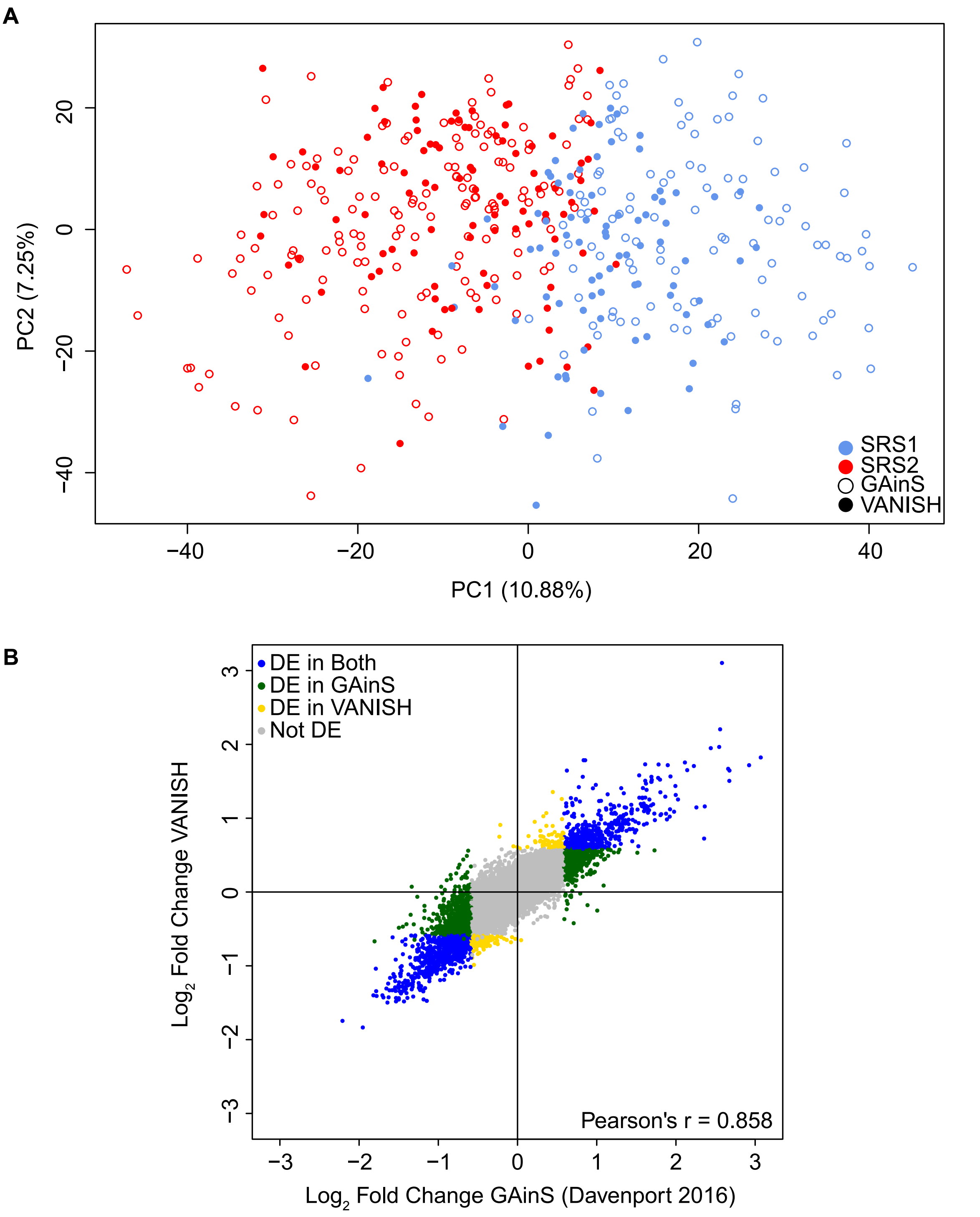

Figure E2. Kaplan-Meier curves comparing survival between SRS1 and SRS2 in those patients randomized to receive A) placebo and B) hydrocortisone. Crosses represent censored patients (n=1 for SRS1 placebo and n=1 for SRS1 hydrocortisone. All other patients were censored at death or day 29.)

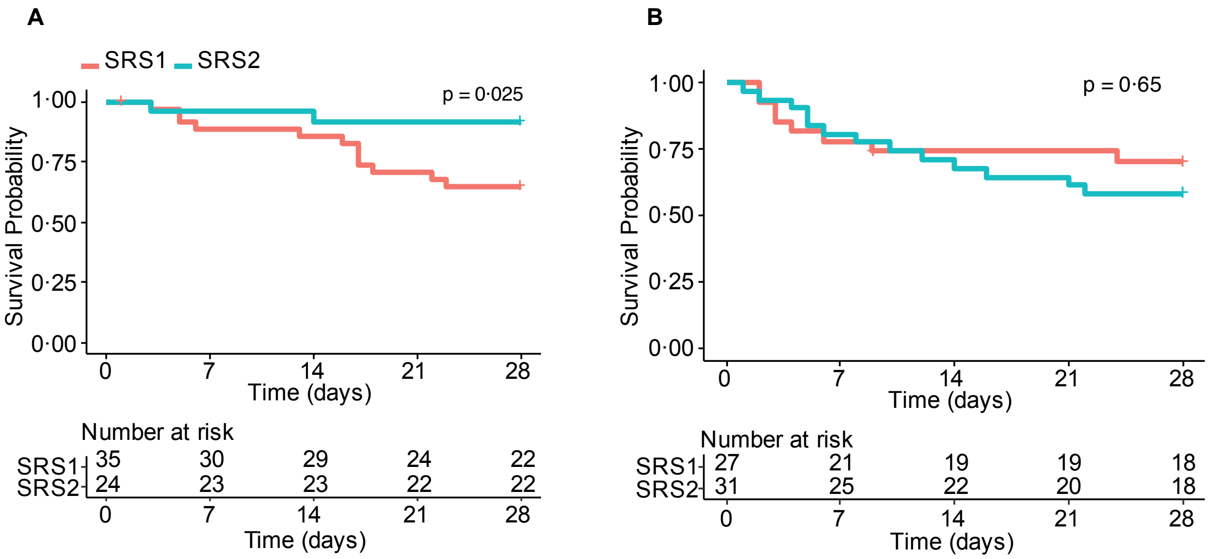

References

1. Bone RC, Balk RA, Cerra FB, Dellinger RP, Fein AM, Knaus WA, Schein RMH, Sibbald WJ. Definitions for sepsis and organ failure and guidelines for the use of innovative therapies in sepsis. Chest 1992;101(6):1644-1655.
2. Huber W, von Heydebreck A, Sultmann H, Poustka A, Vingron M. Variance stabilization applied to microarray data calibration and to the quantification of differential expression. Bioinformatics 2002;18:S96–S104.
3. Johnson WE, Li C, Rabinovic A. Adjusting batch effects in microarray expression data using empirical Bayes methods. Biostatistic*s* 2007;8:118–27.
4. Davenport EE, Burnham KL, Radhakrishnan J, Humburg P, Hutton P, Mills TC, Rautanen A, Gordon AC, Garrard C, Hill AVS, Hinds CJ, Knight JC. Genomic landscape of the individual host response and outcomes in sepsis: A prospective cohort study. Lancet Respir Me*d* 2016;4:259–271.
5. Burnham KL, Davenport EE, Radhakrishnan J, Humburg P, Gordon AC, Hutton P, Svoren-Jabalera E, Garrard C, Hill AVS, Hinds CJ, Knight JC. Shared and distinct aspects of the sepsis transcriptomic response to fecal peritonitis and pneumonia. Am J Respir Crit Care Med 2017;196:328–339.
6. Ritchie ME, Phipson B, Wu D, Hu Y, Law CW, Shi W, Smyth GK. Limma powers differential expression analyses for RNA-sequencing and microarray studies. Nucleic Acids Res 2015;43:e47.
7. Cox, D R; Snell EJ. Analysis of Binary Data, 2nd ed. London: Chapman & Hall; 1989.
8. Salter KC, Fawcett RF. The art test of interaction: A robust and powerful rank test of interaction in factorial models. Commun Stat - Simul Comput 1993;22:137–153.
9. Conover WJ, Iman RL. Rank transformations as a bridge between parametric and nonparametric statistics. Am Stat 1981;35:124–128.

10. Gordon AC, Mason AJ, Thirunavukkarasu N, Perkins GD, Cecconi M, Cepkova M, Pogson DG, Aya HD, Anjum A, Frazier GJ, Santhakumaran S, Ashby D, Brett SJ, VANISH Investigators. Effect of Early Vasopressin vs Norepinephrine on Kidney Failure in Patients With Septic Shock: The VANISH Randomized Clinical Trial. JAMA 2016;316:509–18.
